# A person-centred approach to health promotion for persons 70+ who have migrated to Sweden: promoting aging migrants’ capabilities implementation and RCT study protocol

**DOI:** 10.1186/s12877-015-0005-4

**Published:** 2015-02-14

**Authors:** Susanne Gustafsson, Qarin Lood, Katarina Wilhelmson, Greta Häggblom-Kronlöf, Sten Landahl, Synneve Dahlin-Ivanoff

**Affiliations:** Institute of Neuroscience and Physiology, The Sahlgrenska Academy at the University of Gothenburg, Section for Health and Rehabilitation, Gothenburg, Sweden; University of Gothenburg Centre for Ageing and Health (AgeCap), Gothenburg, Sweden; Centre for Person-Centred Care (GPCC), University of Gothenburg, Gothenburg, Sweden; Department of Public Health and Community Medicine/Social Medicine, Institute of Medicine, the Sahlgrenska Academy at University of Gothenburg, Gothenburg, Sweden

**Keywords:** Aging, Emigrants and immigrants, Health education, Randomised controlled trial, Intervention studies, Activities of daily living, Finland, Balkan Peninsula

## Abstract

**Background:**

There are inequities in health status associated with ethnicity, which may limit older foreign-born persons’ ability to age optimally. Health promotion for older persons who have experienced migration is thus an area of public health importance. However, since research related to this issue is very limited, the study ‘Promoting Aging Migrants’ Capabilities’ was initiated to improve our understanding. The study aims to implement and evaluate a linguistically adapted, evidence-based, health-promoting intervention with a person-centred approach for two of the largest groups of aging persons who have migrated to Sweden: persons from Finland and persons from the Balkan Peninsula.

**Methods/Design:**

This study has a descriptive, analytical, and experimental design. It is both a randomised controlled trial and an implementation study, containing the collection and analysis of both qualitative and quantitative data. The setting is an urban district in a medium-sized Swedish city with a high proportion of persons who were born abroad and whose socio-economic status is low. The intervention comprises four group meetings (‘senior meetings’) and one follow-up home visit made by a multi-professional team. For the randomised controlled trial, the plan is to recruit at least 130 community-dwelling persons 70 years or older from the target group. Additional persons from involved organisations will participate in the study of the implementation. Both the intervention effects in the target group (outcome) and the results of the implementation process (output) will be evaluated.

**Discussion:**

The results of this forthcoming randomised controlled trial and implementation study may be useful for optimising implementation of person-centred, health-promoting initiatives for older persons who have experienced migration. It is also hoped that this combined study will show that the capabilities for optimal aging among older persons born in Finland and the Balkan countries can be improved in the Swedish healthcare context.

**Trial registration:**

The trial was registered at ClinicalTrials.gov April 10, 2013, identifier: NCT01841853.

## Background

Optimal aging may be defined as the capacity to function across many domains (physical, functional, cognitive, emotional, social, and spiritual) to one’s own satisfaction and despite one’s medical conditions [1]. The realization of optimal aging is of vital importance since the proportion of older persons in the population is expected to increase in both Sweden and the rest of Europe [2,3]. Today, persons above 67 years of age constitute about 17% of the Swedish population and are expected to constitute 21% by the year 2020 [3]. Additionally, the number of aging persons who migrate from one country of residence to another is rising [4]. Persons who have migrated to Sweden constitute 13% of the population over 65 years of age, and this figure is expected to increase to 15% by 2020 [3]. According to the parliamentary bill on research [5], the aging population will pose challenges to Swedish health and welfare services, with increased costs for care and healthcare. However, the parliamentary bill also points out that research on aging and health can contribute to great gains, both health-wise and economically, for individuals as well as for society as a whole. In addition, it stresses that the focus should be on using evidence-based knowledge, health promotion, interdisciplinarity, and a person-centred approach [5]. In view of the above information, it is clear that research on the health of older persons who have experienced migration is an area of utmost importance.

The National Board of Health and Welfare in Sweden emphasises that healthcare should systematically integrate and target specific health-promoting and disease-preventive interventions daily to provide equal healthcare for the whole Swedish population [6]. However, aging persons might not have equal opportunities to enjoy good health because of factors at an individual (micro), group (meso), or societal (macro) level, which can limit a person’s capability for optimal aging. Individual factors (e.g. physical health and cognition), group factors (e.g. family and community), and societal factors (e.g. laws and healthcare services) can at any given time form the basis for an individual’s capability set, i.e. what an individual actually can or cannot do [7]. For instance, we know today that there are inequities in health status associated with ethnicity [8], and persons who have migrated to Sweden run a greater risk of developing cardiovascular diseases and mental illness than their native-born counterparts [9,10]. In addition, conceptions of health and aging among persons who were born abroad may be shaped by the process of migration, and thus differ from the ones held by native Swedes [11]. Hence, based on a growing body of knowledge that suggests that age-related decline can be delayed [12], health promotion for older persons could be a strategy for achieving optimal aging. Health promotion is a process that enables people to increase control over and improve their health [13]. It aims to empower individuals by strengthening their capabilities to enhance accomplishment in everyday living and self-efficacy [14]. In targeting equality in the Swedish healthcare context, it is essential to design and evaluate health-promoting initiatives for older persons born abroad.

The persons who would probably benefit most from a health-promoting programme are those who have not yet reached any restrictions in activity level [15], are pre-frail (having less than three of five physical frailty criteria [16]), or are at risk of frailty [17,18]. Frailty is a state of decreased reserve resistance to stressors as a result of cumulative decline across multiple physiological systems, which cause vulnerability to different outcomes [19]. The prevalence of frailty increases with age and is associated with an elevated risk of adverse health outcomes, such as dependence, falls, hospitalisation, institutionalisation, and mortality [19,20]. Older persons who have experienced migration are often excluded from research studies because of linguistic and other barriers [21], limiting the knowledge of healthcare needs within these groups. In addition, they can be at double jeopardy for developing frailty, and are often described as a vulnerable group exposed to isolation and mental illness, as well as having language problems when seeking healthcare services. However, older persons who were born abroad are a heterogeneous group of people, originating from many different countries and with different healthcare needs. Therefore, a person-centred approach to health-promotion, aiming to recognise each person’s different and constantly changing experiences and requirements, is desirable. It can ensure that the persons concerned are able to influence their own healthcare processes, a factor that has been shown to have a significant impact on older persons’ experiences of the overall quality of care [22]. Person-centredness entails shared decision-making, meaning that all decisions concerning care, treatment, rehabilitation, and health-promoting activities ought to be taken in partnership [23].

A literature review and meta-analysis of health-promoting programmes targeting aging persons who have culturally and linguistically diverse backgrounds have shown that research in this area is very limited (Lood Q, Häggblom-Kronlöf G, Dahlin-Ivanoff S: Unpublished observations). To our knowledge, there is no health-promoting programme that integrates a person-centred approach for older persons who were born abroad and are at risk of developing frailty. However, the continuing analyses of a recently completed randomised controlled trial (RCT), ‘Elderly Persons in the Risk Zone’ [24], is providing growing evidence that a health-promoting programme targeting pre-frail persons 80 years and older can achieve good results. Two different interventions have been evaluated: 1) a single preventive home visit and 2) a group-based intervention with four senior meetings and one follow-up home visit. The senior meetings show the most advantageous results, including delayed deterioration in self-rated health, dependence in activities of daily life (ADL), and morbidity [25-27]. Further, qualitative interviews show that this intervention is perceived as a key to action, supporting self-change towards healthier life style choices [28]. The hypothesis in ‘Elderly Persons in the Risk Zone’ was that if an intervention is conducted when older persons are pre-frail, then it is possible to prevent or delay deterioration in health. This hypothesis, and the design and content used in the evidence-based ‘senior meetings’ intervention (with minor adaptation), ought to apply to older pre-frail persons born abroad.

Consequently, the present study, ‘Promoting Aging Migrants’ Capabilities’, was designed, aiming to implement and evaluate the aforementioned evidence-based, health-promoting intervention (‘senior meetings’) with a person-centred approach for two of the largest groups of aging persons who have migrated to Sweden; persons from Finland and persons from the Balkan Peninsula [29]. In preparation for the study, interviews were held with older persons who have experienced migration, and focus groups were conducted with personnel working in the targeted urban district [30,31]. Additionally, collaboration was initiated with reference groups of older persons from the target group living in the city in question to gather knowledge and provide guidance on how to adapt the original intervention protocol to older persons in the target population.

The implementation of a new method or approach in a healthcare organisation may face resistance or various difficulties; it is often not a simple and straightforward process. Therefore, various factors need to be studied and assessed, such as usability, adaptations, barriers, fidelity, and anticipated impact. To broaden the perspective, the present study will evaluate both intervention effects in the target group (outcome) and the results of the implementation process (output). To fully understand the intervention and implementation process, a mix of methods will be used. This approach will hopefully contribute to improving the capabilities of older persons who are born abroad to age optimally in the Swedish welfare context. This paper presents the design of the Promoting Aging Migrants’ Capabilities Study, an RCT as well as an implementation study, which was written in accordance with the SPIRIT guidelines for study protocol content [32,33], and the CONSORT recommendations for reporting pragmatic RCTs [34].

### Aims and research questions for the study

The overall aim of the Promoting Aging Migrants’ Capabilities Study is to implement the evidence-based ‘senior meetings’ intervention among older persons who have migrated to Sweden, a person-centred approach to health promotion to support optimal aging in the Swedish context. The study aims to prove the following two hypotheses:

1. If a health-promoting programme is introduced when older persons who were born abroad are pre-frail, it is possible to prevent or delay deterioration in health (i.e. dependence in ADL, self-rated health) and life satisfaction.
2. The design and content of the evidence-based senior meetings can be used in the context of older persons who have migrated to Sweden.

Specifically, the study addresses the following research questions:

1. Can a person-centred, health-promoting programme for older persons who were born abroad:
  Prevent decline in physical function, activity performance, leisure pursuits, and life satisfaction?
  Be a supportive factor in the social network, and if so, how?
  Have an impact on the consumption of care?
2. Could the design and content of the evidence-based senior meetings:
  Be adapted for older persons who have migrated to Sweden, and if so, how?
  Support self-change behaviour, and if so, how?
  Be implemented with high fidelity?
3. How do older persons who were born abroad, and the personnel in the targeted urban district, experience the programme, its significance, and importance for health?

## Methods/Design

The Promoting Aging Migrants’ Capabilities Study has a descriptive, analytical, and experimental design. It is both an RCT and an implementation study, containing the collection and analysis of both qualitative and quantitative data. The combination of quantitative and qualitative methods in the study will best answer the research questions, as well as taking advantage of the strengths of both methodological approaches [35]. The participants in the RCT are randomised into two study arms (one intervention and one control group), and outcomes at 6- and 12-month follow-ups will be analysed. A pilot study has been conducted before the full-scale RCT to assess the feasibility of the programme, and has guided the final protocol (Lood Q, Gustafsson S, Dahlin Ivanoff S: Unpublished observations). The implementation part of the study has a case study design [36], and will be carried out alongside the RCT. In addition, individual in-depth interviews of both participants and personnel will be conducted to determine the perceived effect and significance of the programme.

### Study context

Activities in care and healthcare for older persons in Sweden include a number of actors that interact in different ways. Medical care is predominantly performed in the public sector (80%), and healthcare costs are financed mainly through taxes and government grants. Cities and municipalities are responsible for services for older persons. The aim of these services is to ensure that older persons are able to live as independently as possible in their own homes. When older persons are no longer able to manage daily life independently, they can apply for assistance from the municipal home help service. The extent of such support is subject to an assessment of needs; it may include meals on wheels, help with cleaning and shopping, assistance with personal care, safety alarms, transportation services, and healthcare.

The location of the Promoting Aging Migrants’ Capabilities Study is an urban district in a medium-sized city in the western part of Sweden. The urban district is situated outside the city centre, but within city limits, with the majority of living accommodations consisting of blocks of flats. In 2013, the total population was 49,920 persons of which 5,491 (11%) were 65 years or older [37]. Fifty per cent of all inhabitants in the urban district were born in countries outside Sweden (other Nordic countries as well as countries within and outside Europe). The most frequently represented countries of birth, regardless of the person’s age, were in descending order: Iraq, Iran, Finland, Bosnia-Herzegovina, and the former Yugoslavia. The latter three were, however, dominant countries of birth for persons 65 years or older. There are no available statistics of causes of immigration for residents in the targeted urban district, but these causes would likely mirror those for Sweden as a whole: work/studies, escape from war, family ties, and other reasons. In 2012, the general education level and income level of residents in the urban district were lower, and the sickness rate higher, than in the population of the city as a whole [37].

### Intervention

The intervention implemented in the Promoting Aging Migrants’ Capabilities Study is the evidence- and group-based, health-promoting ‘senior meetings’ programme. The main purpose of the senior meetings is to facilitate discussion of the aging process and to provide tools and strategies to enable participants to solve various problems that may arise at home so that they can remain living at home in a safe and secure way. A booklet containing different aspects of health self-management (e.g. physical activity, medication, nutrition, assistive devices, adaptation of housing, memory, quality of life) is used as material for group discussions [38]. The senior meetings also inform participants of what the municipality provides in the form of local meeting places, activities run by local associations, physical training for seniors, walking groups, and possibilities of offering or accepting help on a voluntary basis. Available help and support offered by the municipality are addressed and discussed. Identification of risks for falls and advice on how to prevent them are also included. For an overview of the chapters in the booklet and the profession responsible for each section, see Table 1.

**Table 1:** **Themes from the booklet used in senior meetings in the promoting aging migrants’ capabilities study**

**Themes from the booklet**	**Principal professional** *
Aging	PT
Physical activity helps keep you physically fit	PT
Food is a prerequisite for health	PT
You can take care of problems with your health	RN
How to use medicines	RN
To cope with everyday life	OT
You do not need to feel insecure	OT
Technology in everyday life	OT
Will I lose my memory?	OT
Life events and quality of life during aging	SW
Anyone who needs help can get help	SW

*Physiotherapist (PT), registered nurse (RN), occupational therapist (OT), and social worker (SW).

The senior meetings are conducted by an operative group consisting of professionals employed in the urban district: an occupational therapist, a registered nurse, a physiotherapist, and a social worker. All professionals receive education and training on the programme content, and on leading groups prior to study commencement. The programme comprises four weekly senior meetings in small groups (four to six participants), in addition to an individual follow-up home visit two to three weeks after the last senior meeting. Having groups enables the possibility of peer education with participants learning from each other [39], and a person-centred approach increases the possibilities for participants to be seen as experts on their own situation [40]. Respect for the participant and his/her values and giving each participant the opportunity to maintain and develop his/her control over his/her own everyday activities, is essential in the meetings.

Some minor but important adaptations of the original senior meetings protocol [24] were made when designing the Promoting Aging Migrants’ Capabilities Study. First, since several languages were to be involved, the study adopted a bilingual approach, where all written participant material is printed in Swedish and the participants’ mother tongue. Recordings of material to compact disc (CD) in participants’ preferred languages are also available. If needed and sought, interpreters are engaged in study activities. Second, based on the dialogue with the reference groups, a section covering how to handle post-traumatic stress in everyday life was added to the booklet. Third, when the booklet was revised, some smaller sections that were deemed redundant by personnel in the urban district were removed to reduce the text. Finally, to further clarify and consolidate the person-centred approach, seminars based on scientific literature and dialogue were held jointly with the operative group and research group.

In this intervention, the risk of causing adverse events is judged to be low. However, any unforeseen harm will be documented by the operative group and reported to both the research group and the steering committee. When necessary, adequate responsive actions, for instance referral to medical care, will also be taken. To promote comparability of RCT study groups, concomitant care and interventions during participation in the study will be avoided as much as possible. Participants randomised to the control group will not be included in the intervention, but will receive conventional care on their own initiative, as well as undergoing study baseline and follow-up assessments.

### Design of the RCT

A brief structured summary of the RCT part of the Promoting Aging Migrants’ Capabilities Study is provided in Table 2.

**Table 2:** **A brief structured summary of the RCT part of the promoting aging migrants’ capabilities study** *

**Data category**	**Information**
Primary registry and trial identifying number	ClinicalTrials.gov NCT01841853
Date of registration in primary registry	10 April 2013
Secondary identifying numbers	-
Source(s) of monetary or material support	The Swedish Research Council, the Centre for Person-Centred Care (GPCC) at Gothenburg University, Sweden
Primary sponsor	The University of Gothenburg, Sweden
Secondary sponsor(s)	The City of Gothenburg, Sweden
	The Region of Västra Götaland, Sweden
Contact for public queries	SDI, synneve.dahlin-ivanoff@.gu.se
Contact for scientific queries	SDI, synneve.dahlin-ivanoff@.gu.se, the Sahlgrenska Academy at the University of Gothenburg, Institute of Neuroscience and Physiology/Section for Health and Rehabilitation, Sweden
Public title	The Promoting Aging Migrants’ Capabilities Study
Scientific title	The Promoting Aging Migrants’ Capabilities Study: a person-centred approach to health promotion for persons 70+ who have migrated to Sweden
Countries of recruitment	Sweden
Health condition(s) or problem(s) studied	Health promotion for older persons born abroad
Intervention(s)	Intervention: 4 senior meetings and 1 follow-up home visit
	Control: Conventional care and follow-up
Key inclusion and exclusion criteria	Ages eligible for study: ≥ 70 years; Sexes eligible for study: both; Accepts healthy volunteers: Yes
	Inclusion criteria: adult person (≥70 years); born in Finland or any of the four countries in the Balkan Peninsula; living in an urban district in a medium-sized city; living in ordinary housing; not dependent on informal or formal help in daily activities
	Exclusion criteria: impaired cognition [Mini Mental State Examination (MMSE) below 80% of administered items]
Study type	Interventional
	Allocation: randomised; Intervention model: parallel assignment; Masking: non-blind
	Primary purpose: Health promotion
	Phase III
Date of first enrolment	08 August 2012
Target sample size	130
Recruitment status	Recruiting
Primary outcome	Activities of Daily Living (ADL)
Key secondary outcomes	Frailty indicators, falls and fear of falls, symptoms, depression, healthcare consumption, self-rated health, life satisfaction, leisure activities, assistive technology, and social support

*WHO Trial Registration Data Set.

#### Study population

The intention is that the study group should comprise as representative a sample as possible of older persons in the urban district who have migrated to Sweden. For pragmatic reasons, particularly considering the need for language resources, we chose to include persons from two of the largest immigrant groups in the targeted urban district, one Nordic country (Finland) and one European region (the Balkan Peninsula, including the countries Bosnia-Herzegovina, Croatia, Montenegro, and Serbia, the populations who share their mother tongue). Persons eligible for the RCT must therefore comply with all of the following criteria for randomisation: born in Finland or any of the selected four countries in the Balkan Peninsula (Bosnia-Herzegovina, Croatia, Montenegro, and Serbia); 70 years of age or older; living in ordinary housing in the urban district; and not dependent on informal or formal help in daily activities. The only exclusion criterion is impaired cognition (Mini Mental State Examination (MMSE) below 80% of administered items [41]).

#### Outcomes

Activities of daily living (ADL) is the primary outcome measure [42,43]. Secondary outcome measures are: physical frailty indicators (mobility, strength, balance, cognition, nutrition, fatigue, physical activity, and visual impairment), falls and fear of falls, symptoms, depression, healthcare consumption, self-rated health, life satisfaction, leisure activities, assistive technology, and social support. The outcomes have, to a large extent, been tested for validity and reliability. All outcome measures are to be assessed at baseline and at the 6- and 12-month follow-ups. Details are provided in Table 3.

**Table 3:** **Outcome measurements and follow-ups in the RCT part of the promoting aging migrants’ capabilities study**

**Primary outcome**	**Measurement**	**T0**	**T1**	**T2**
		**baseline**	**6-months**	**12-months**
Activities of Daily Living (ADL)	The ADL-staircase	X	X	X
**Secondary outcome**	**Measurement**			
Fatigue	The Mob-T scale	X	X	X
Grip strength	North Coast-dynamometer	X	X	X
Physical activity	Questionnaire	X	X	X
	Physical and domestic activity scale	X	X	X
Balance	The Berg balance scale	X	X	X
Gait speed	Four-meter walking test	X	X	X
Weight loss	The Göteborg Quality of Life Instrument	X	X	X
Cognition	Mini Mental State Examination (MMSE)	X	X	X
Visual impairment	KM-visual acuity chart	X	X	X
Falls	Questionnaire	X	X	X
Fear of falls	Questionnaire	X	X	X
Symptoms	The Göteborg Quality of Life Instrument	X	X	X
Depression	GDS 20	X	X	X
Healthcare consumption	Register data	X	X	X
Self-rated health	SF 36 (one question)	X	X	X
Life satisfaction	Fugl-Meyer -LiSat	X	X	X
Participation/Leisure activities	Questionnaire	X	X	X
Assistive technology	Questionnaire	X	X	X
Social support	Questionnaire	X	X	X

#### Recruitment, participant timeline, and randomisation

**Figure 1:**
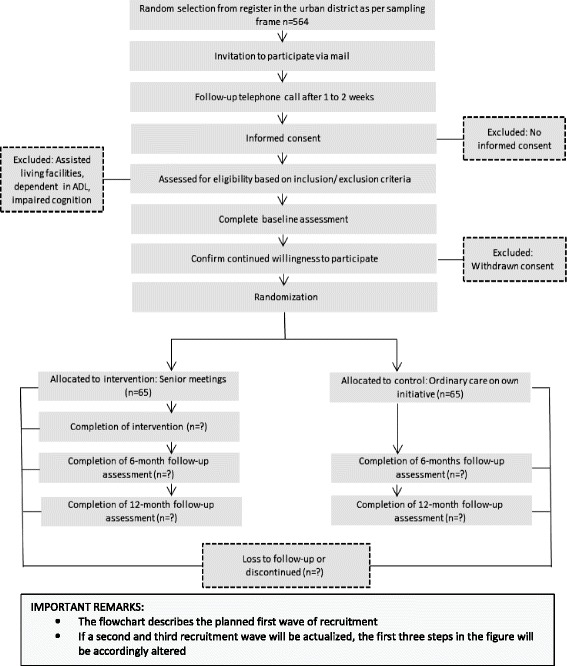
**Planned flow of participants in the Promoting Aging Migrants’ Capabilities Study.**

The results of the pilot study preceding this full-size RCT (Lood Q, Gustafsson S, Dahlin Ivanoff S: Unpublished observations) indicated that the recruitment procedure described in the original protocol for the senior meetings [24] needed to be adjusted to be more effective. Therefore, a plan consisting of three waves of recruitment strategies was adopted. The waves are hierarchical and an appropriate wave was to be added if an antecedent wave was unsuccessful in reaching the target number for inclusion. The first wave implies that participants are drawn at random from official registers in the urban district. All addresses, equally divided between the populations (Finland and the four countries in the Balkan Peninsula), are listed in random order (two separate lists). Based on the sampling lists, participants will be consecutively included in the study until the decided sample size is reached (n≥65 from each population). Letters will be sent to all randomly selected persons asking them to participate in the study. Invited participants also receive a description of the study, how it is to be conducted, and what is expected of those consenting to participate. The letter stresses the fact that participation is voluntary; it is followed by a telephone call about 1–2 weeks later. If no telephone number is to be found in public records, a second letter with a request to make contact by telephone is sent. During the call, the persons will be informed verbally about the study and given the opportunity to ask questions if anything is unclear. They are also asked personally if they would like to participate, while again stressing that participation is voluntary. Everyone who fulfils the criteria for inclusion and decides to participate is randomised by the research assistant after baseline assessment to one of the two groups using sealed, opaque envelopes prepared by an unattached researcher. Participants living together in the same household are allocated to the same group. Wave two is identical to wave one but involves an expansion of the targeted area to also include another adjacent urban district with similar demographics. Finally, wave three entails word-of-mouth promotion through one-chain snowballing [44]. This strategy uses former study participants and key persons in the aforementioned reference groups to facilitate spreading information about the study. Interested persons fulfilling inclusion criteria are invited to contact the operative group for further information and to undergo baseline assessment when appropriate. The third wave also includes local radio station advertisements containing brief information in the participants’ mother tongue on the study and how to contact the operative group. A detailed overview of the planned flow of participants is provided in Figure 1.

The invitation process and data collection are conducted by trained research assistants and conducted in the language preferred by the participant. The standardised baseline assessment comprises an interview, measurements, and observation. Follow-up data, identical to the baseline assessment except for demographics, will be collected in both groups at 6- and 12 months after the intervention. The complete data collection form, as well as consent and participant information forms, can be retrieved by contacting the first author (SG) or last author (SDI). In addition, information on reasons for any non-adherence (e.g. discontinuation of intervention) and non-retention (i.e. consent withdrawn or lost to follow-up) will be collected. Data from municipal registers will also be obtained and used to study care consumption.

The recruitment of participants began in autumn 2012 and is planned to be completed by the end of December 2014. The intervention began at the same time as the inclusion, and will be completed no later than 6 months after the last inclusion. The 12-months follow-up will be completed in January 2016. A detailed study timeline is shown in Table 4.

**Table 4:** **Promoting aging migrants’ capabilities study timeline**

**Period:**	**2009-2012**	**2012/Q1 – Q2**	**2012/Q3 – Q4**	**2013/Q1 – Q2**	**2013/Q3 – Q4**
**Activities:**	Establish contacts	Formation of research organisation	Training of personnel	Recruitment of participants	Recruitment of participants
	Pre-planning	Establishing study protocol	Recruitment of participants	Intervention	Intervention
	Formation of steering		Intervention	Data collection	Data collection
	committee		Data collection	Pilot study	Pilot study publication
**Period**	**2014/Q1 – Q2**	**2014/Q3 – Q4**	**2015/Q1 – Q2**	**2015/Q3 – Q4**	**2016/Q1 – Q2**
**Activities:**	Recruitment of participants	Recruitment of participants	Data collection	Data collection	Article writing
	Intervention	Intervention	Data analyses	Data analyses	Dissemination of results
	Data collection	Data collection	Article writing	Article writing	
	Study protocol publication	Data analyses		Reporting to funding agencies	
		Article writing			

#### Masking (blinding)

Because of the nature of the intervention, neither participants nor the professionals in the urban district can be blinded to allocation in the RCT. In addition, supported by the pilot study results, assessors cannot be blinded since we need to match assessors and participants linguistically. Finally, we also assume that there would be less attrition if the participants could meet the same assessor at most of the follow-ups.

#### Power calculation

The power calculation was based on results gained in the preceding study ‘Elderly Persons in the Risk Zone’ [24], using the Berg balance scale as a basis. The Berg balance scale is one of the secondary outcome variables (range 0–56), with assumed mean values for the intervention and control groups of 32 and 28, respectively (15% difference), and a standard deviation of 8 in both groups. To be able to detect a significant difference between the two groups in a two-sided test with a significance level of 0.05 and 80% power, we need at least 65 persons in each group. Therefore, a total of at least 130 persons will be included.

#### Data management

All participant information, including informed consent forms, interviews on audio files, etc., will be stored separately from study records identified by code number in locked file cabinets in areas with limited access. The original data collection forms are to be stored in numerical order in a secure and accessible place and manner for a period of 10 years after completion of the study. An external research assistant will enter data into the computer and also perform quality control of the database. During and after the study completion, the research group will have access to the full database. Any modifications to data written to the database will be documented in a log book linked to the database. Regular back-ups of the database will be undertaken automatically by the university’s information technology department. The main database, as well as local databases, will be secured with password-protected access systems.

No data monitoring committee will be needed since risks in connection with this study are minimal, there is a fixed target figure for included participants, and no interim analyses are planned. Even so, the research group, and ultimately the research leader, will be responsible for database security and quality.

#### Statistical analyses

Analyses of intervention outcomes and change over time will be made on the basis of the intention-to-treat principle, which means that participants will be analysed on the basis of the group to which they were randomised [45]. Standard adjustment of analyses according to eventual differences between groups at baseline will be made if necessary. After a thorough examination of the characteristics of collected data, the research group will consider different options for handling missing data. One possibility is the imputation method used in the preceding study ‘Elderly Persons in the Risk Zone’ [25,26], where a missing value was imputed with a value based on the median change of deterioration (MCD) between two measuring points (baseline and respective follow-up) of all those who participated at both measuring points. However, other methods of handling missing data will also be considered, and a final decision will be made by the lead researcher in consultation with a renowned statistician. Regardless of the method chosen, sensitivity analyses will be performed to compare the results to complete case analyses [46]. Although it is desirable, no subgroup analyses are predetermined. Instead, a valuation of the quality of collected data will determine if and which of these analyses are possible. Both descriptive and analytical statistics will be used to compare the groups and to measure change over time. Non-parametric statistics will be used in all cases where ordinal data are to be analysed; otherwise, parametric statistics will be used. A *p-*value of 0.05 or less will be considered significant. Statistical analyses will be performed using IBM SPSS Statistics for Windows, Version 22.0 (Armonk, NY, USA: IBM Corp. Released 2013).

### Design of the implementation study

#### Theoretical basis

**Figure 2:**
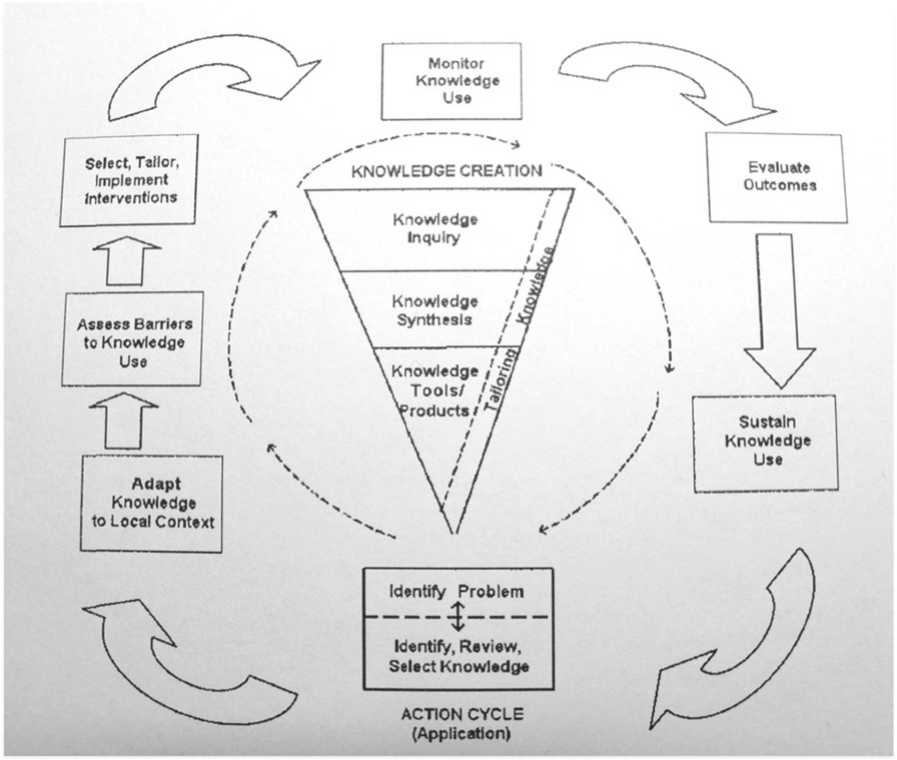
**The knowledge-to-action model [** 47 **].**

The implementation in any given context can be seen as a dynamic process involving several phases. The knowledge-to-action model (KTA) [47] forms an overarching theoretical framework for the implementation process, which involves all stakeholders (i.e. researchers, decision-makers, and users) as they cooperatively transform an intervention into practice. The goal of the KTA model is to enhance health status through knowledge creation and action, both of which consist of ideal phases (Figure 2). The focus, as regards the aim of this study, is the action cycle (application), where the evidence-based senior meetings intervention is implemented into practice. In this practice, it is important to identify components that support or inhibit the implementation. To evaluate the impact in an implementation results in new knowledge, which can form a basis for creating useful and practically applicable strategies, tools, and products to be used in similar contexts in the future.

#### Study population

In addition to participants recruited for the RCT part of the study, members of the steering committee (five persons), the research group (nine persons), and the operative group (four persons) will be included in the population for the implementation study.

#### Data collection and analysis

Several aspects of the senior meeting implementation will be studied: the feasibility of the recruitment method; its effectiveness; hindrances at all levels (personal, professional, organisational); eventual adaptations of the recruitment method and intervention; and intervention fidelity. It is essential to report that even though the Promoting Aging Migrants’ Capabilities Study uses a predetermined intervention protocol, it entails possibilities for adaptation by answering to any contextual needs. For instance, the recruitment of participants will be closely monitored with openness to adaptation by the development of new strategies, as well as strategies for participant adherence to intervention and follow-ups. Any adaptation to the intervention protocol is decided, documented, and evaluated in collaboration with the operative group, research group, and steering committee at regular scheduled audits throughout the study. Further, an evaluation of whether the design and content of the senior meetings support self-change by participants will be conducted. As recommended for case studies, multiple sources of data collection will be used, thereby enabling triangulation [36]. Data will be collected throughout the study; methods include direct observations, qualitative interviews, questionnaires, and study documentation. Data analysis will be done in accordance with guidance given for each distinct data collection method, and thereafter aggregated for final results.

### Qualitative studies

In addition to the study of intervention effects and implementation output, it is important to find out how participants and personnel in the urban district experience different aspects of the health-promoting programme. This will be done using individual interviews. These interviews will give the respondents opportunities to highlight their priorities in their own way and may reveal ideas that are not anticipated by the researchers [48]. In addition, the complex factors that affect habits and ways of thinking about health in the context of migration will be illuminated.

#### Data collection and analysis

Individual interviews, which may be characterized as semi-structured ‘in-depth’ interviews, will be carried out with study participants and personnel in the urban district. The interviews aim to elicit the individual experiences and perceptions of participating in a person-centred and health-promoting intervention. We will consider the balance between male and female study participants. The interviews will be audio-taped, transcribed verbatim, and analysed, to identify patterns and themes in the statements [49,50]. The qualitative data will enable an analysis of similarities and differences between the perceptions and experiences of participants and personnel at both the individual and group level. Results of the qualitative studies may contribute important knowledge to the evaluation of the study as a whole.

### Ethical considerations

The ethical problems that may arise in the study primarily concern those who receive intervention versus those persons who receive conventional care. If the intervention proves effective and has a positive effect on the health and wellbeing of participants, then it will be offered to everyone in the study control group once the study has been completed. In addition, there is a risk of experiencing questions in the data collection form as tiring or bothersome, which was also addressed in the pilot study (Lood Q, Gustafsson S, Dahlin Ivanoff S: Unpublished observations). Overall, the risk is small compared with the benefit that participants will gain from obtaining information and support (the intervention group) and regular assessment and discussion of their own capacity. Nevertheless, the researchers will be observant of signs that may indicate fatigue, and in such cases will make the visit short and return on a later occasion. If, however, a participant experiences substantial difficulties coping or has a temporary lack of motivation, and this becomes a threat to continued participation, a revised version of the data collection form with a few prioritised items will be offered as an alternative to the full version.

The risk of stigmatisation of persons born abroad and ethical dilemmas about dividing persons based on age and country of birth were carefully weighed against the importance of visualising inequities between different groups of people. Health and healthcare inequities due to age and migration status will never be bridged if researchers continue to exclude aging persons who are born abroad or who do not speak the dominant language. Regarding language, inclusion of persons who have a different mother tongue from the researchers requires specific ethical consideration. It is always important to ensure that everyone who participates is aware of what they consent to, and when dealing with linguistic differences, consideration must be given to linguistic preferences of participants. In the present study, all participants were able to choose the language they preferred to be addressed in. The Regional Ethical Review Board in Gothenburg approved this study (reference #821-11). The final approval document, as well as the informed consent form to be signed by participants, can be retrieved by contacting the first author (SG) or the last author (SDI).

### Study sponsors and organisation

The Promoting Aging Migrants’ Capabilities Study is a collaborative study between three sponsors in western Sweden: the Sahlgrenska Academy at the University of Gothenburg, the city of Gothenburg, and the region of Västra Götaland. The study organisation consists of a steering committee with representatives of the sponsors and the two other parties in the organisation (the research group and the operative group in the targeted urban district). Representation on the steering committee enables the three sponsors to play a role in agreement of the final study protocol and review of study progress. The sponsors have no role, however, in the analyses and interpretation of study data or the decision to submit results. For detailed information on the members of each respective organisational party and its area of responsibility, see Table 5.

**Table 5:** **Study organisation parties in the promoting aging migrants’ capabilities study and respective area of responsibility**

**Steering committee**	**Research group**	**Operative group**
• The lead researcher, professor, also representing the University, SDI	• A lead researcher, professor, SDI	• A team leader of the operative group, a social worker
• A senior consultative researcher, professor, SL	• One senior researcher, KW	• A registered occupational therapist
• A representative of the targeted city	• Two junior researchers, SG, GHK	• A registered physiotherapist
• A representative of the health department of the targeted region	• Four doctoral students	• A registered nurse
• The team leader of the operative group	• One research assistant	
Organising steering committee meetings	Study planning	Preparation of brochure with study information
Agreement on final study protocol	Study design and conduct	Recruitment of participants
Reviewing progress of study and if necessary agreeing changes to the protocol	Ethics committee applications	Conducting intervention
Budget administration and decision	Preparation of study protocol and revisions	
	Randomisation	
	Baseline assessment	
	Responsible for study master database	
	Data verification	
	Maintenance of study information technology system and data entry	
	Follow-up data collection	
	Publication of study reports	

### Study protocol amendments

Any modifications to the protocol that may impact the conduct of the study or potential benefit of the participant, including changes in study objectives, study design, population, sample sizes, study procedures, or significant administrative aspects, will require a formal amendment to the protocol. Such amendment will be agreed upon by the steering committee, and approved by the Regional Ethical Review Board in Gothenburg prior to implementation. Administrative changes to the protocol are minor corrections and/or clarifications that have no effect on the way the study is to be conducted. These administrative changes will be agreed upon by the steering committee, and will be documented in meeting records.

### Dissemination policy

Research articles with different focuses (e.g. the intervention outcome, the implementation output, and qualitative papers) will be submitted to relevant scientific journals. Individuals who fulfil authorship criteria should not remain hidden and should have authority over manuscript content. Similarly, those who do not fulfil such criteria should not be granted authorship. No professional writer will be employed in the writing of articles, but language validators will be engaged. The research findings are planned to be presented at appropriate national and international conferences. Information about results relevant to politicians, policy makers, and relevant personnel will be made at scheduled meetings. In addition, information for participants and the public will be given at public meetings in easily accessible premises in the targeted urban district, starting not later than 1 year after completion of the study. Popular science reports and summary research information in the participants’ mother tongue will also be compiled and made publically available.

For transparency, upon written request from recognized researchers in the field, the study’s lead researcher will consider sharing deidentified data. Such actions will be decided in line with the policy at the University of Gothenburg, and will be considered no later than 3 years after the collection of the 1-year post-randomisation interviews.

## Discussion

It is critical that every clinical study has a complete and transparent protocol, which can facilitate trial conduct and appraisal by communicating relevant information to key stakeholders. In response, the present study protocol of the Promoting Aging Migrants’ Capabilities Study addresses all recommendations for the minimum relevant protocol items to include in a study protocol as recommended by SPIRIT [32,33]. In addition, the RCT part of our study is written in accordance with recommendations for reporting pragmatic randomised controlled trials [34]. However, no guideline for how to report implementation or qualitative studies has been found, which explains why these parts of the study protocol have a more free composition. Even so, by providing this composite information, we intend to adequately convey the complexity of the design of this combined RCT and implementation study.

In designing the Promoting Aging Migrants’ Capabilities Study, we address older persons who have migrated to Sweden, which is a growing proportion of the older population. The older persons who have experienced migration are an under-prioritised group for which more research is needed not only to throw light on their need for medical and social care (and how their needs may best be met), but also to implement already-existing evidence-based knowledge within the health service, welfare, and special services.

Health-promoting interventions among older persons can lead to great gains, both health-wise and economically, for both the individual and society. They can prevent the onset of injury and illness and support optimal aging. Specifically, we expect this study to show positive effects on ADL, self-rated health, and life satisfaction, and to be cost-effective in terms of lower costs for healthcare and social services. We also anticipate that the implementation will contribute important knowledge about how to transfer an evidence-based, person-centred, health-promoting programme from one context to another. In addition, the qualitative studies will highlight important aspects of the participants’ experiences of the intervention, its relevance to health, and their perception of what can be effective. Overall, the results may be useful for optimising the implementation of person-centred, health-promoting initiatives and for improving the capabilities for optimal aging among older persons born in Finland and countries in the Balkan Peninsula in the Swedish healthcare context.

## Abbreviations

RCT: Randomised controlled trial
ADL: Activities of daily life
SPIRIT: Standard protocol items: recommendations for interventional trials
CONSORT: Consolidated standards of reporting trials
MMSE: Mini mental state examination
MCD: Median change of deterioration
KTA: Knowledge to action
PT: Physiotherapist
RN: Registered nurse
OT: Occupational therapist
SW: Social worker

## References

[1] Brummel-Smith K (2007). Optimal aging, part I: demographics and definitions. Ann Longterm Care.

[2] European Union. The 2012 ageing report: underlying assumptions and projection methodologies. European Economy 4|2011 [PDF] 2011. http://europa.eu/epc/pdf/2012_ageing_report_en.pdf. Accessed 21 dec 2014.

[3] Statistics Sweden (2009). Demographic Reports 2009:1, The Future Population of Sweden 2009–2060.

[4] Phillipson C (2007). The ‘elected’ and the ‘excluded’: sociological perspectives on the experience of place and community in old age. Ageing Soc.

[5] Sveriges regering. Regeringens proposition 2012/13:30 - Forskning och innovation. [PDF] 2012. http://www.regeringen.se/content/1/c6/20/13/68/ab3950ad.pdf. Accessed 21 dec 2014.

[6] Socialstyrelsen (2009). Vård och omsorg om äldre, lägesrapport 2008.

[7] Sen A (2009). The Idea of Justice.

[8] Vårdanalys. En mer jämlik vård är möjlig-Analys av omotiverade skillnader i vård, behandling och bemötande. [PDF] 2014. http://www.vardanalys.se/Global/Rapporter%20pdf-filer/2014/2014-7-En%20mer%20j%c3%a4mlik%20v%c3%a5rd%20%c3%a4r%20m%c3%b6jlig_webb.pdf. Accessed 21 dec 2014.

[9] Silveira E, Skoog I, Sundh V, Allebeck P, Steen B (2002). Health and well-being among 70-year-old migrants living in Sweden - Results from the H 70 gerontological and geriatric population studies in Göteborg. Soc Psych Psych Epid.

[10] Statens folkhälsoinstitut. Födelselandets betydelse, om hälsan hos olika invandrargrupper i Sverige. Stockholm: Statens Folkhälsointitut;2002. Rapport/Statens folkhälsoinstitut; 2002:29.

[11] Torres S (2001). Understandings of successful ageing in the context of migration: the case of Iranian immigrants in Sweden. Ageing Soc.

[12] Fries JF (2005). The compression of morbidity. Milbank Q.

[13] World Health Organisation [WHO] (2001). International Classification of Functioning, Disability and Health (ICF).

[14] Tengland PA (2008). Empowerment: a conceptual discussion. Health Care Anal.

[15] Gustafsson S, Edberg A-K, Johansson B, Dahlin-Ivanoff S (2009). Multi-component health promotion and disease prevention for community-dwelling frail elderly persons: a systematic review. Eur J Ageing.

[16] Fried LP, Ferrucci L, Darer J, Williamson JD, Anderson G (2004). Untangling the concepts of disability, frailty, and comorbidity: implications for improved targeting and care. J Gerontol A: Biol Med Sci.

[17] Bouman A, Van Rossum E, Nelemans P, Kempen GIJM, Knipschild P (2008). Effects of intensive home visiting programs for older people with poor health status: a systematic review. BMC Health Serv Res.

[18] Vass M, Avlund K, Lauridsen J, Hendriksen C (2005). Feasible model for prevention of functional decline in older people: municipality-randomized, controlled trial. J Am Geriatr Soc.

[19] Fried LP, Tangen CM, Walston J, Newman AB, Hirsch C, Gottdiener J (2001). Frailty in older adults: evidence for a phenotype. J Gerontol A: Biol Med Sci.

[20] Rockwood K (2005). Frailty and its definition: a worthy challenge. J Am Geriatr Soc.

[21] Hussain-Gambles M, Atkin K, Leese B (2004). Why ethnic minority groups are under-represented in clinical trials: a review of the literature. Health Soc Care Community.

[22] McCormack B, Mitchell EA, Cook G, Reed J, Childs S (2008). Older persons’ experiences of whole systems: the impact of health and social care organizational structures. J Nurs Manag.

[23] Ekman I, Swedberg K, Taft C, Lindseth A, Norberg A, Bring E (2011). Person-centered care - ready for prime time. Eur J Cardiovasc Nurs.

[24] Dahlin-Ivanoff S, Gosman-Hedström G, Edberg A-K, Wilhelmson K, Eklund K, Zidén L (2010). Elderly persons in the risk zone. Design of a multidimensional, health-promoting, randomised three-armed controlled trial for “prefrail” people of 80+ years living at home. BMC Geriatr.

[25] Gustafsson S, Eklund K, Wilhelmson K, Edberg A-K, Johansson B, Kronlöf GH (2013). Long-term outcome for ADL following the health-promoting RCT–elderly persons in the risk zone. Gerontologist.

[26] Gustafsson S, Wilhelmson K, Eklund K, Gosman-Hedström G, Zidén L, Kronlöf GH (2012). Health-promoting interventions for persons aged 80 and older are successful in the short term-results from the randomized and three-armed elderly persons in the risk zone study. J Am Geriatr Soc.

[27] Behm L, Wilhelmson K, Falk K, Eklund K, Zidén L, Dahlin-Ivanoff S (2014). Positive health outcomes following health-promoting and disease-preventive interventions for independent very old persons: long-term results of the three-armed RCT elderly persons in the risk zone. Arch Gerontol Geriatr.

[28] Behm L, Zidén L, Dunér A, Falk K, Dahlin-Ivanoff S (2013). Multi-professional and multi-dimensional group education–a key to action in elderly persons. Disabil Rehabil.

[29] Statistiska centralbyrån (Statistics Sweden). Befolkningsstatistik. [Database] 2014. http://www.statistikdatabasen.scb.se/pxweb/sv/ssd/?rxid=4ccce9b5-1566-428a-87ea-6e5c8b956069. Accessed 21 dec 2014.

[30] Lood Q, Dahlin-Ivanoff S, Dellenborg L, Mårtensson L. Health-promotion in the context of ageing and migration: a call for person-centred integrated practice*.* Int J Integr Care. 2014. Jan-Mar; NL:UI:10-1-114771. 10.5334/ijic.1162 PMC3943616 24605072

[31] Lood Q, Häggblom-Kronlöf G, Dellenborg L. Embraced by the past, hopeful for the future: meaning of health to ageing persons who have migrated from the Western Balkan region to Sweden*.* Aging Soc. 2015. In press.

[32] Chan AW, Tetzlaff JM, Altman DG, Laupacis A, Gøtzsche PC, Kreza-Jeric’ K (2013). SPIRIT 2013 statement: defining standard protocol items for clinical trials. Ann Intern Med.

[33] Chan AW, Tetzlaff JM, Gøtzsche PC, Altman DG, Mann H, Berlin JA (2013). SPIRIT 2013 explanation and elaboration: guidance for protocols of clinical trials. BMJ.

[34] Zwarenstein M, Yreweek S, Gagnier JJ, Altman DG, Tunis S, Haynes B (2008). Improving the reporting of pragmatic trials: an extension of the CONSORT statement. BMJ.

[35] Burke Johnson R, Onwuegbuzie AJ (2004). Mixed methods research: a research paradigm whose time has come. Educ Research.

[36] Yin RK, Nilsson B (2007). Fallstudier: design och genomförande.

[37] Göteborg Stad (The city of Gothenburg). Statistik Göteborg. [Web Page] 2014. http://www4.goteborg.se/prod/G-info/statistik.nsf. Accessed 21 dec 2014.

[38] Vårdalinstitutet (The Swedish Institute for Health Sciences), S. Dahlin-Ivanoff (Ed.) Livslots för seniorer [PDF] 2009. http://www.vardalinstitutet.net/sites/default/files/tr/naring/naringdocs/litteraturdocs/8916.pdf. Accessed 21 dec 2014.

[39] Shiner M (1999). Defining peer education. J Adolescence.

[40] Leplege A, Gzil F, Cammelli M, Lefeve C, Pachoud B, Ville I (2007). Person-centredness: conceptual and historical perspectives. Disabil Rehabil.

[41] Folstein MF, Folstein SE, McHugh PR (1975). ‘Mini mental state’. A practical method for grading the cognitive state of patients for the clinician. J Psych Res.

[42] Sonn U, Grimby G, Svanborg A (1996). Activities of daily living studied longitudinally between 70 and 76 years of age. Disabil Rehabil.

[43] Sonn U, Åsberg KH (1991). Assessment of activities of daily living in the elderly. A study of a population of 76-year-olds in Gothenburg, Sweden. Scand J Rehabil Med.

[44] Sadler GR, Lee H, Lim RS, Fullerton J (2010). Recruiting hard-to-reach United States population sub-groups via adaptations of snowball sampling strategy. Nurs Health Sci.

[45] Altman DG (1999). Practical Statistics for Medical Research.

[46] Bennett A (2001). How can I deal with missing data in my study?. Aust NZ J Publ Heal.

[47] Graham ID, Logan J, Harrison MB, Straus SE, Tetroe J, Caswell W (2006). Lost in knowledge translation: time for a map?. J Contin educ Health.

[48] Dahlin IS, Hultgren J (2006). Understanding the multiple realities of everyday life: basic assumptions in focus group methodology. Scand J Occup Ther.

[49] Charmaz K (2004). Premises, principles, and practices in qualitative research: revisiting the foundations. Qual Health Res.

[50] Malterud K (2012). Systematic text condensation: a strategy for qualitative analysis. Scand J Public Health.

